# Diet, Nutrition, and Rhinosinusitis: A Systematic Review of Dietary Interventions and Exposures

**DOI:** 10.3390/nu18142299

**Published:** 2026-07-14

**Authors:** Cenorina Martinez, Emily M. Sitkowski, Maria E. Martinez, Esbeyda Martinez, Priyali N. Patel, Hannah L. Walsh, Sammy Khalili

**Affiliations:** 1 Intent Medical Group, Endeavor Health Advanced Neurosciences Center, Northwest Community Hospital, Arlington Heights, IL 60005, USA; emily.sitkowski@endeavorhealth.org (E.M.S.); priyali.patel@endeavorhealth.org (P.N.P.); hlwalsh14@gmail.com (H.L.W.); sammy.khalili@endeavorhealth.org (S.K.); 2 College of Arts and Sciences, Cornell University, Ithaca, NY 14853, USA; mem542@cornell.edu; 3 Diane Tyrrell Department of Nursing and Public Health, Elmhurst University, Elmhurst, IL 60126, USA; emart9390@365.elmhurst.edu

**Keywords:** chronic rhinosinusitis, rhinosinusitis, sinonasal inflammation, sinonasal inflammatory disease, diet, dietary patterns, nutrition, anti-inflammatory diet, dietary exposures, food-based interventions

## Abstract

**Background/Objectives**: Rhinosinusitis (RS) is a prevalent inflammatory condition with substantial morbidity and variable response to standard therapies. Diet is a modifiable determinant of systemic inflammation, yet its role in sinonasal disease remains incompletely defined. **Methods**: A systematic review was conducted in accordance with Preferred Reporting Items for Systematic Reviews and Meta-Analyses guidelines. PubMed, Scopus, Cochrane Library, and Web of Science were searched from inception through 28 June 2026. Studies assessing dietary exposures or interventions in RS were included using predefined criteria. Risk of bias was assessed using ROBINS-I and Joanna Briggs Institute tools. Due to heterogeneity in study design and outcomes, findings were synthesized narratively. **Results**: Ten studies met inclusion criteria (5 adult, 4 pediatric, 1 mixed), including interventional and observational designs. Anti-inflammatory dietary patterns were associated with improved sinonasal outcomes. In children, Mediterranean diet adherence reduced recurrent inflammatory episodes, and reduced sugar intake improved sinus infection scores with decreased TNF-α concentrations. In adults, both allergen-guided elimination diets and a low-arachidonic acid, low-salicylate, high-fiber dietary intervention improved symptom severity, with accompanying improvements in endoscopic outcomes. Observational studies demonstrated that higher fruit intake was associated with lower RS prevalence, whereas higher fat intake, caloric intake, Dietary Inflammatory Index scores, frequent meals prepared away from home, and ultra-processed food intake were associated with increased disease risk or sinonasal symptom burden. Phenotype-specific associations included increased dietary salicylate sensitivity and food-triggered symptom exacerbation among patients with nasal polyposis. **Conclusions**: Dietary patterns and specific nutritional exposures are associated with RS outcomes across age groups. Anti-inflammatory dietary profiles demonstrate potential protective effects, whereas pro-inflammatory dietary patterns are associated with increased disease burden. However, the predominance of observational data and study heterogeneity limit causal inference. Well-designed prospective and randomized studies are needed to define the therapeutic role of dietary modification in RS.

## 1. Introduction

Rhinosinusitis (RS) is an inflammatory disorder of the paranasal sinuses classified as acute (<4 weeks), subacute (4–12 weeks), chronic (>12 weeks), or recurrent (≥4 episodes/year) [1,2]. Common symptoms include nasal congestion, rhinorrhea, facial pressure or pain, hyposmia, sneezing, and nasal obstruction [3,4].

Chronic rhinosinusitis (CRS) is associated with substantial quality-of-life impairment, increased healthcare utilization, and significant economic burden [5,6]. Management includes medical therapy, including intranasal corticosteroids and saline irrigation, with systemic corticosteroids and antibiotics used when indicated. Endoscopic sinus surgery is reserved for patients with refractory disease [3,7].

Despite these interventions, CRS frequently persists and exhibits variable treatment response, indicating that potentially modifiable contributors to disease may be involved. CRS is increasingly recognized as a biologically heterogeneous disorder comprising distinct inflammatory endotypes (type 1, type 2, and type 3), each characterized by differing immune profiles, clinical behavior, and therapeutic responsiveness [8]. Since dietary composition is known to modulate low-grade systemic inflammation through effects on cytokine signaling, oxidative stress, and immune-metabolic pathways [9,10,11], it is plausible that diet-mediated reductions in systemic inflammatory burden may improve sinonasal symptoms.

A prior narrative review proposed dietary modification as a nonpharmacologic strategy in refractory CRS, noting the absence of direct evidence and calling for systematic evaluation [12]. Accordingly, this systematic review evaluates associations between dietary patterns, dietary interventions, and food- or nutrient-based exposures and the development, severity, and recurrence of RS. The objective is to clarify the current state of evidence and assess whether dietary modification may represent a clinically relevant adjunctive strategy in the management of RS.

## 2. Materials and Methods

### 2.1. Data Sources and Search Strategy

This systematic review followed the Preferred Reporting Items for Systematic Reviews and Meta-Analyses (PRISMA) guidelines. The protocol was registered in PROSPERO (CRD420251184931). PubMed, Scopus, the Cochrane Library, and Web of Science were searched from inception through 28 June 2026. Reference lists of all included studies were manually screened to identify additional relevant articles. Searches were limited to English-language human studies, with no restrictions on publication date.

The search was guided by the Population, Intervention, Comparison, Outcome, Study Design (PICOS) framework. Populations included children, adolescents, and adults with diagnostically or symptom-defined RS. Both adult and pediatric populations were included given overlapping inflammatory mechanisms and symptom-based diagnostic criteria across age groups. Interventions and exposures included dietary modifications, dietary patterns, and food- or nutrient-based exposures. Comparators reflected usual dietary intake or alternative dietary patterns when applicable. Outcomes include sinonasal disease development, presence, severity, recurrence, or symptom burden. Both interventional and observational study designs were eligible. Full search strategies are provided in Supplementary Table S1.

Studies were eligible if they evaluated dietary exposures or interventions related to sinonasal outcomes consistent with RS. Eligible conditions included CRS, chronic rhinosinusitis with nasal polyps (CRSwNP), chronic rhinosinusitis without nasal polyps (CRSsNP), allergic fungal rhinosinusitis (AFRS), chronic sinusitis, and symptom-defined RS phenotypes, including persistent nasal congestion or rhinorrhea or RS occurring as an inflammatory complication (IC). Symptom-defined RS was included to capture pediatric and population-based studies in which objective confirmation was not routinely performed.

Studies were required to report a defined dietary exposure and at least one sinonasal outcome. Eligible designs included randomized and nonrandomized interventional studies, cohort studies, cross-sectional studies, and case–control studies. Non-human studies, non-English publications, and publications without primary data were excluded, as were reviews, case reports, abstracts, editorials, and book chapters. For synthesis, studies were grouped by dietary exposure category and outcome domain, with stratification by study design.

### 2.2. Study Selection and Data Extraction

Two reviewers independently screened titles and abstracts and reviewed full-text articles to determine eligibility, with discrepancies resolved by consensus. Data from all included studies were independently extracted using a standardized form. Study investigators were not contacted for additional or confirmatory data. No automation tools were used at any stage of screening or data extraction.

Outcomes prespecified for data extraction include disease presence or development, disease severity, symptom burden, recurrence, and frequency of infectious or inflammatory episodes. For each outcome domain, all results reported by each study that were compatible with the outcome definition were extracted, including patient-reported symptom measures, and clinician-assessed outcomes. Additional variables collected included study design, sample size, and population characteristics.

### 2.3. Assessment of Study Quality

Risk of bias was independently assessed by two reviewers. Prospective nonrandomized studies were evaluated using the Risk Of Bias in Non-randomized Studies of Interventions (ROBINS-I) tool, assessing bias across seven domains and assigning overall judgments of low, moderate, serious, or critical risk [13]. Cross-sectional and case–control studies were appraised using Joanna Briggs Institute (JBI) critical appraisal checklists, evaluating study design, outcome measurement validity, confounding, and statistical analysis [14,15]. Discrepancies were resolved by consensus. No automation tools were used in the quality assessment process.

### 2.4. Effect Measures and Synthesis Methods

Inclusion of both pediatric and adult populations introduced anticipated age-related clinical heterogeneity, which was addressed through narrative synthesis. Additional heterogeneity in study design, dietary exposures, and outcomes precluded quantitative meta-analysis. Effect measures were synthesized descriptively as reported, including mean differences, odds ratios, percent change, and frequency-based outcomes. Studies were assigned to prespecified narrative synthesis groups by dietary exposure category and stratified by interventional versus observational design. Data were summarized as reported, without transformation, imputation, or conversion of summary statistics. No subgroup analyses, sensitivity analyses, or certainty of evidence were conducted due to the absence of quantitative synthesis.

## 3. Results

### 3.1. Study Selection and Characteristics

**Figure 1:**
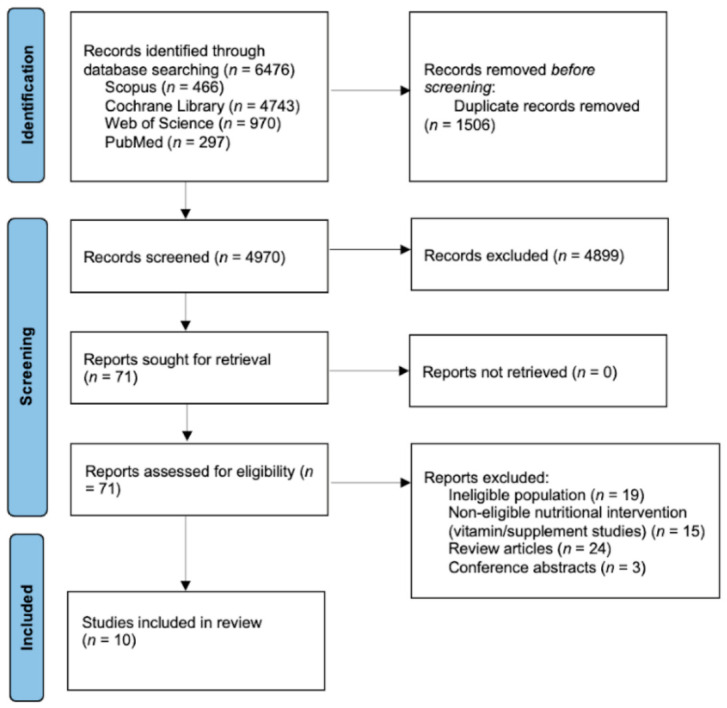
PRISMA flow diagram illustrating the systematic literature search and study selection process.

The PRISMA flow diagram summarizing the study selection process is presented in Figure 1. A total of 6476 records were identified through database searching. After removal of 1506 duplicate records, 4970 records remained for title and abstract screening. Of these, 4899 records were excluded based on title and abstract review. The full texts of 71 articles were assessed for eligibility, of which 61 were excluded because of an ineligible population, ineligible dietary intervention or exposure, review article publication type, or conference abstract publication. Ultimately, ten studies met the inclusion criteria, including five cross-sectional studies, one prospective case–control study, three prospective interventional studies, and one retrospective nonrandomized comparative cohort study. Detailed study characteristics are presented in Table 1 and Table 2. Detailed information on the confounding variables included in the multivariable models for each observational study is provided in Supplementary Table S5.

### 3.2. Quality Assessment in Studies

**Figure 2:**
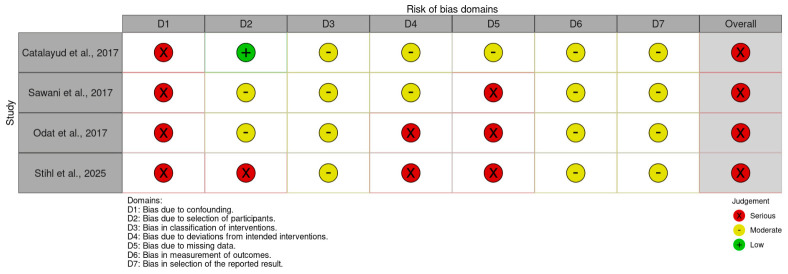
Risk of bias assessment for the included non-randomized studies [16,17,18,19] using the ROBINS-I tool, presented as a traffic light plot generated with the robvis package (version 0.3.1) [13].

Non-randomized interventional studies demonstrated an overall serious risk of bias according to the ROBINS-I assessment [16,17,18,19]. The observational studies were of moderate to high methodological quality based on the JBI Critical Appraisal Checklists, with common limitations related to exposure and outcome ascertainment [20,21,22,23,24,25]. Risk-of-bias assessments are summarized in Figure 2 and Table 3 and Table 4. Detailed domain-level assessments for each study are provided in Supplementary Tables S2–S4.

### 3.3. Dietary Interventions and Sinonasal Outcomes (Pediatric)

Evidence from two prospective interventional studies suggests that dietary modification may improve pediatric sinonasal outcomes [16,17]. Adherence to a traditional Mediterranean diet for one year was associated with a marked reduction in recurrent inflammatory episodes, with the mean number of episodes decreasing from 4.64 ± 0.70 at baseline to 0.70 ± 0.90 at follow-up (mean difference −3.94 ± 0.84; 95% CI −4.08 to −3.74; *p* < 0.001) [16]. At study completion, 54% of participants experienced no inflammatory episodes, whereas 25% and 16% reported one and two episodes, respectively. Similarly, reducing sugar-sweetened beverage consumption over two weeks significantly improved sinus infection scores compared with controls (*p* = 0.005) [17]. Serum TNF-α concentrations also decreased significantly (Z = −2.38; *p* = 0.02), whereas IL-10 demonstrated a non-significant upward trend (Z = 1.80; *p* = 0.07). No significant changes were observed in nasal obstruction (*p* = 0.08), IL-1β, or IL-6.

### 3.4. Dietary Interventions and Sinonasal Outcomes (Adult)

Among adults with CRS, two studies evaluated elimination and anti-inflammatory dietary interventions [18,19]. An allergen-guided elimination diet significantly improved Lund-Kennedy (LK) symptom scores at six weeks (*p* = 0.008) and 12 weeks (*p* = 0.002), with corresponding improvements in endoscopic scores (*p* = 0.04 and *p* = 0.008, respectively) [18]. Five participants achieved complete endoscopic resolution (LK score = 0) at the final follow-up, although improvements across individual symptom domains did not reach statistical significance. Likewise, a structured low-arachidonic acid, low-salicylate, high-fiber diet combined with individualized dietary counseling significantly improved SNOT-22 scores from 39.5 ± 11.0 at baseline to 30.3 ± 10.2 at three months and 24.2 ± 7.8 at six months (*p* < 0.001) [19]. Mean Visual Analogue Scale (VAS) symptom scores decreased from 5.6 ± 1.1 to 4.6 ± 1.0 and 3.5 over the same period (*p* < 0.001), while endoscopic polyp scores improved from 4.1 ± 1.9 to 1.8 ± 1.4 (*p* < 0.0001). Brief Smell Identification Test scores increased from 5.8 ± 1.6 to 6.9 ± 1.9 but did not reach statistical significance.

### 3.5. Dietary Quality and Rhinosinusitis Prevalence (Pediatric and Adolescent)

Evidence regarding dietary quality in pediatric RS was limited to one population-based study [24]. Children with RS reported more frequent consumption of salty snacks and sugar-sweetened carbonated beverages than those without RS (*p* = 0.007 and *p* = 0.023, respectively). After multivariable adjustment, low fruit intake (<5–6 servings/week) was independently associated with increased odds of RS (OR 1.75; 95% CI 1.07–2.86; *p* = 0.027), as was frequent salty snack consumption (≥1–2 times/week; OR 1.91; 95% CI 1.14–3.20; *p* = 0.014).

### 3.6. Dietary Quality and Rhinosinusitis Prevalence (Adult)

Associations between overall dietary quality and CRS prevalence were examined in two population-based cross-sectional studies [20,22]. Greater dietary fat intake (IQR = 34.085; *p* = 0.001), higher total energy intake (IQR = 981.106; *p* = 0.004), and greater dietary inflammatory potential were associated with increased odds of CRS, with participants in the highest Dietary Inflammatory Index (DII) quartile demonstrating higher odds than those in the lowest quartile (OR 1.484; 95% CI 1.132–1.946; *p* = 0.004) [22]. Conversely, higher overall fruit intake was associated with lower odds of CRS in both unadjusted (OR 0.75; 95% CI 0.58–0.96) and adjusted analyses (aOR 0.73; 95% CI 0.55–0.97), whereas potato consumption remained independently associated with increased odds after multivariable adjustment (aOR 1.82; 95% CI 1.03–3.23) [20]. The association between tropical fruit intake and CRS was no longer significant after adjustment.

### 3.7. Contemporary Dietary Behaviors and Sinonasal Outcomes (Adult)

Contemporary dietary behaviors, including away-from-home (AFH) meal consumption and ultra-processed food (UPF) intake, were evaluated in two large population-based studies comprising 58,583 adults [23,25]. Compared with consuming fewer than one AFH meal per week, adjusted odds of CRS were higher among individuals consuming 1–4 meals per week (aOR 1.139; 95% CI 1.029–1.260; *p* = 0.0119) and ≥5 meals per week (aOR 1.210; 95% CI 1.078–1.358; *p* = 0.0013) [23]. Greater AFH meal frequency was also associated with persistent olfactory dysfunction but not with EPOS 2020-defined endoscopic findings [23]. Similarly, participants in the highest quartile of UPF intake had increased odds of self-reported sinusitis (OR 1.54; 95% CI 1.15–2.05; *p* = 0.007), dysgeusia (OR 1.79; 95% CI 1.15–2.80; *p* = 0.02), and xerostomia (OR 2.26; 95% CI 1.68–3.02; *p* < 0.001) [25]. The association with dysgeusia was attenuated after adjustment for xerostomia (OR 1.62; *p* = 0.07), whereas no significant associations were observed with hyposmia, allergic rhinitis, hay fever, allergy-related nasal congestion, or serum total IgE concentrations.

### 3.8. Phenotype-Specific Dietary Sensitivity Outcomes (Adult)

Dietary salicylate sensitivity across CRS phenotypes was examined in one multicenter case–control study [21]. Overall, 19.1% of participants with CRS reported dietary salicylate sensitivity. After adjustment for age, sex, and aspirin sensitivity, salicylate-related symptom exacerbation was more likely among participants with CRSwNP (aOR 3.16; 95% CI 1.78–5.61; *p* < 0.001) and CRSsNP (aOR 2.03; 95% CI 1.15–3.58; *p* = 0.01), with the strongest association observed in CRSwNP. These findings remained unchanged after excluding participants with autoimmune disease, immunodeficiency, or ciliary dyskinesia. Participants with nasal polyposis, particularly those with AFRS, also reported higher frequencies of food-triggered symptom exacerbation, most notably following wine consumption (controls 0.9%, CRSwNP 18.4%, AFRS 44.0%; *p* < 0.001) and nut consumption (AFRS 16.0% vs. controls 0%; *p* = 0.001).

## 4. Discussion

This systematic review synthesized the available evidence on the relationship between dietary interventions, dietary quality, and dietary behaviors and RS across pediatric and adult populations. Overall, the findings suggest that diet may influence both the development and clinical course of RS. Across diverse study designs, anti-inflammatory dietary patterns were generally associated with improvements in sinonasal symptoms, endoscopic findings, and inflammatory outcomes [16,17,18,19], whereas Western dietary exposures such as greater inflammatory potential, higher fat and energy intake, sugar-sweetened beverages, UPFs, and frequent AFH meals were associated with greater disease prevalence or symptom burden [20,21,22,23,24,25]. Although the evidence remains limited by methodological heterogeneity and the predominance of observational studies, the consistency of findings across multiple dietary domains supports diet as a potentially modifiable factor that may complement existing strategies for the prevention and management of RS.

A notable finding is that diverse dietary interventions converged toward a common therapeutic principle despite substantial differences in composition. Mediterranean dietary patterns [16], allergen-guided elimination diets [18], and structured anti-inflammatory interventions [19] each produced clinically meaningful improvements in disease-specific outcomes, including symptom severity, endoscopic findings, and quality of life. Observational studies likewise demonstrated that healthier dietary behaviors, such as greater fruit consumption, were associated with lower odds of RS [20], whereas Western dietary characteristics were associated with greater disease burden [20,21,22,23,24,25]. Collectively, these findings suggest that overall dietary quality, rather than individual nutrients or foods, may be more relevant to sinonasal health, consistently distinguishing dietary patterns that promote or attenuate systemic inflammation.

This convergence is biologically plausible. CRS is characterized by persistent mucosal inflammation, epithelial barrier dysfunction, oxidative stress, and dysregulated innate and adaptive immune responses. Diets rich in fruits, vegetables, whole grains, legumes, and unsaturated fats provide antioxidants, polyphenols, fiber, vitamins, and omega-3 fatty acids that reduce oxidative stress, preserve epithelial barrier integrity, and suppress pro-inflammatory cytokine production [26,27]. Conversely, diets high in saturated fats, refined carbohydrates, and UPFs have been associated with increased systemic inflammation, altered immune regulation, and disruption of epithelial barrier function [28,29]. Dietary composition may also influence systemic immune responses through diet-microbiome interactions, though this pathway has not been directly examined in RS. Taken together, these mechanisms provide a plausible framework through which dietary modification may influence sinonasal inflammation.

Our findings are consistent with broader nutrition literature demonstrating the anti-inflammatory effects of healthy dietary patterns. Previous systematic reviews have reported that adherence to Mediterranean and other anti-inflammatory patterns is associated with lower circulating concentrations of inflammatory biomarkers, including CRP, TNF-α, and IL-6 [26], while diets with greater inflammatory potential are associated with increased systemic inflammation [28,29]. The present review extends these observations to RS across both pediatric and adult populations. One pediatric intervention study demonstrated significant reductions in serum TNF-α following reduced sugar-sweetened beverage consumption, providing preliminary mechanistic support for the clinical improvements observed [17]. However, no significant changes were observed for IL-1β or IL-6, and improvements in olfactory function did not reach statistical significance, suggesting that dietary modification may not influence all inflammatory pathways equally [17].

Intervention studies demonstrated substantially greater improvements in clinical outcomes than would be expected from the modest associations reported by population-based studies. Mediterranean adherence markedly reduced recurrent inflammatory episodes in young children [16], while anti-inflammatory and elimination interventions significantly improved disease-specific quality of life and endoscopic findings among adults with CRS [18,19]. In contrast, observational studies generally reported weak-to-moderate associations, with adjusted odds ratios typically ranging between approximately 0.73 and 1.91 [20,21,22,23,24,25]. This discrepancy likely reflects differences in study design and populations. Intervention studies enrolled symptomatic individuals with established disease, allowing dietary modification to influence active inflammatory processes, whereas observational studies evaluated habitual exposures across broader community populations in whom diet represents only one of many contributing factors. Nevertheless, the agreement in the direction of findings strengthens the hypothesis that dietary quality contributes to sinonasal health.

Several methodological considerations warrant careful interpretation. Reverse causality remains plausible for some observed associations. Individuals with CRS commonly experience olfactory dysfunction, altered taste perception, and reduced enjoyment of food, all of which may influence dietary preferences and encourage greater consumption of highly palatable foods rich in salt, sugar, or fat [30]. Patients with persistent symptoms may also rely more heavily on convenience or processed foods because of reduced appetite or diminished quality of life [31]. Poorer dietary quality may therefore represent a consequence of RS rather than a causal determinant, and because most included studies were cross-sectional, the temporal relationship between dietary exposures and disease onset cannot be established.

Age-related immune and sinonasal maturation is another important consideration when interpreting the pediatric intervention studies. Young children experience progressive maturation of innate and adaptive immune function during the first years of life, accompanied by increasing immunologic memory following repeated exposure to respiratory pathogens [32]. Anatomical development of the upper airway and paranasal sinuses may also contribute to age-related changes in susceptibility to recurrent respiratory infections [33]. Improvements observed over time may therefore be partially attributable to normal developmental changes. Although the magnitude of improvement following Mediterranean intervention exceeds what maturation alone would typically produce [16], future randomized controlled trials with appropriate comparator groups are needed to distinguish treatment effects from developmental trajectories.

Residual confounding remains an important limitation of the observational literature. Although most studies adjusted for demographic and lifestyle variables, healthier dietary patterns frequently coexist with other health-promoting behaviors, including greater physical activity, lower smoking prevalence, higher socioeconomic status, and improved healthcare access [34]. Individuals adhering to healthier diets may also seek medical attention earlier or comply more readily with treatment, introducing health-consciousness bias that statistical adjustment cannot fully eliminate [34,35]. Because the observed associations were generally modest, they remain particularly susceptible to residual confounding. The evidence should therefore be interpreted as demonstrating an association between dietary quality and RS rather than a causal relationship.

The evidence also suggests that dietary effects may differ across CRS phenotypes. One study reported significantly greater dietary salicylate sensitivity among individuals with CRS with nasal polyps, with the strongest associations in patients with AFRS, who also reported more frequent food-triggered symptom exacerbations, particularly following wine consumption [21]. However, these findings should be interpreted cautiously because both dietary salicylate exposure and food-triggered symptom exacerbation were assessed using non-validated, self-reported questionnaires, introducing the potential for recall bias and exposure and outcome misclassification. Allergen-guided elimination diets similarly improved symptom severity and endoscopic findings among selected patients [18], and a structured low-arachidonic acid, low-salicylate intervention conducted exclusively in patients with nasal polyps was associated with improvements in symptom burden and endoscopic polyp scores [19]. Overall, the evidence supporting phenotype-specific dietary responsiveness remains limited and should be considered hypothesis-generating until confirmed by higher-quality prospective studies. Nevertheless, these preliminary findings suggest that dietary modification may not confer uniform benefit and that specific inflammatory endotypes or allergic phenotypes may demonstrate greater responsiveness. This concept aligns with the recognized heterogeneity of CRS and highlights the potential role of precision nutrition as an adjunct to phenotype-directed medical management.

A further observation is the limited use of objective mechanistic outcomes. While several interventions demonstrated meaningful improvements in symptom burden and endoscopic severity, few simultaneously evaluated inflammatory biomarkers or other biological correlates of response. The reduction in serum TNF-α following decreased sugar-sweetened beverage consumption provides preliminary evidence that dietary modification may directly influence inflammatory pathways [17], though the absence of significant changes in other cytokines underscores the complexity of CRS pathophysiology. Future studies incorporating comprehensive inflammatory profiling, epithelial barrier markers, microbiome analyses, and objective olfactory assessments will be essential to characterize the biological mechanisms underlying these observations.

This review has several strengths. To our knowledge, it is the first systematic review to comprehensively evaluate both dietary interventions and habitual dietary exposures in relation to RS across pediatric and adult populations. By synthesizing interventional and observational evidence, it provides a broad overview of the current literature while identifying consistent dietary themes that may influence sinonasal health. The review extends beyond individual nutrients to evaluate overall dietary patterns, contemporary behaviors, and phenotype-specific sensitivities, and its inclusion of clinically relevant outcomes such as symptom severity, disease-specific quality of life, endoscopic findings, inflammatory biomarkers, and disease prevalence strengthens the clinical applicability of the findings.

Limitations of this systematic review should be considered. The interventional evidence was constrained by design weaknesses, including reliance on before-and-after designs without parallel control groups, small exploratory samples, and substantial participant attrition, all of which limit the strength of the conclusions that can be drawn. In pediatric populations, improvements observed over time may also partly reflect age-related immune maturation rather than the dietary intervention itself. Much of the remaining evidence was observational and predominantly cross-sectional, precluding causal inference, and substantial heterogeneity across study populations, RS definitions, dietary assessment methods, intervention protocols, follow-up durations, and outcome measures prevented quantitative meta-analysis. Dietary intake was predominantly self-reported using food frequency questionnaires or dietary recalls, introducing potential for recall bias and exposure misclassification. Several individual dietary exposures were also difficult to interpret in isolation, particularly where preparation methods that substantially alter nutrient composition and inflammatory potential were not accounted for, underscoring the value of characterizing overall dietary patterns rather than single foods. We did not contact the original study authors to obtain missing data, and few studies incorporated objective inflammatory biomarkers or mechanistic outcomes, so this review was limited to the published literature and offers limited insight into the underlying biological pathways. Given these limitations, the current evidence should be considered hypothesis-generating rather than definitive.

Future research should prioritize adequately powered randomized controlled trials evaluating standardized dietary interventions using validated RS outcome measures, objective endoscopic assessments, inflammatory biomarkers, and microbiome analyses. Longer follow-up is needed to determine the durability of treatment effects and whether dietary modification reduces disease recurrence, medication requirements, or the need for surgery. Investigations should also examine dietary interventions according to phenotype and inflammatory endotype, including CRSwNP, CRSsNP, aspirin-exacerbated respiratory disease, and AFRS, to determine whether personalized nutritional strategies improve clinical outcomes. Prospective longitudinal studies are needed to clarify the temporal relationship between dietary quality and disease development while minimizing reverse causality and residual confounding.

## 5. Conclusions

The available evidence demonstrates a consistent association between dietary inflammatory burden and RS across diverse populations and study designs. Dietary patterns characterized by greater inflammatory potential were generally associated with increased disease burden, whereas dietary patterns with lower inflammatory potential and preliminary dietary interventions were associated with more favorable clinical outcomes. Although the current evidence is insufficient to establish causality or support routine dietary modification as standard therapy for RS, the convergence of epidemiologic observations, early interventional findings, and biologically plausible mechanisms suggests that diet may represent a modifiable contributor to the multifactorial pathogenesis of the disease.

## Figures and Tables

**Table 1:** Characteristics of included interventional studies.

Study	Population	Design/Setting	Disease Type	Dietary Intervention	Outcomes	Primary Findings
Catalayud et al., 2017 [16]	Children aged 12–33 months (*n* = 128; 65 boys, 63 girls)	Prospective, before-and after comparison study	Acute RS as an IC of URTI (IC outcome component)	Traditional Mediterranean diet for 1 year	IC episodes, URTI episodes, antibiotic use	IC episodes decreased from 4.64 to 0.70 after 1 year (mean difference −3.94; *p* < 0.001); 54% had no episodes at follow-up.
Sawani et al., 2017 [17]	Children aged 2–6 years (*n* = 11 completed; 8 intervention, 3 control)	Prospective, nonrandomized cohort pilot study	RS-like symptoms (symptom-defined): daily nasal congestion or rhinorrhea ≥ 3 months, without acute illness	Reduced sugar intake for 2 weeks	SN-5 sinus infection score, TNF-α, IL-1β, IL-6	Reduced sugar-sweetened beverage intake improved sinus infection scores vs. controls (*p* = 0.005) and decreased TNF-α (*p* = 0.02); no significant change in IL-1β or IL-6.
Odat et al., 2017 [18]	Adults with refractory CRS (*n* = 16 analyzed; 22 enrolled)	Prospective, open-label study	Refractory CRS	Food elimination therapy for 6 weeks	LK symptom score, LK endoscopic score	LK symptom scores improved at 6 weeks (*p* = 0.008) and remained improved at 12 weeks (*p* = 0.002); LK endoscopic appearance scores also improved at 6 weeks (*p* = 0.040) and 12 weeks (*p* = 0.008), with complete endoscopic resolution in 5 of 16 patients.
Stihl et al., 2025 [19]	Adults (*n* = 45; 15/45 dietary intervention)	Retrospective nonrandomized comparative cohort study	CRSwNP	Low-arachidonic acid, low-salicylate, high-fiber diet with dietary counseling (follow-up at 3 and 6 months)	SNOT-22, VAS, endoscopy (polyposis), BSIT	SNOT-22 scores decreased from 39.5 to 24.2 (*p* < 0.001), VAS scores decreased from 5.6 to 3.5 (*p* < 0.001), and polyposis scores decreased from 4.1 to 1.8 (*p* < 0.0001) after 6 months; objective smell scores increased from 5.8 to 6.9 but were not significantly different.

IC, inflammatory complication; TNF-α, tumor necrosis factor alpha; IL-1β, interleukin-1 beta; IL-6, interleukin-6; LK, Lund–Kennedy; SNOT-22, 22-item Sinonasal Outcome Test; VAS, Visual Analog Scale; BSIT, Brief Smell Identification Test; RS, rhinosinusitis; Acute RS, acute rhinosinusitis; CRS, chronic rhinosinusitis; CRSwNP, chronic rhinosinusitis with nasal polyps; URTI, upper respiratory tract infection.

**Table 2:** Characteristics of included observational studies.

Study	Population	Design/Setting	Disease Type	Dietary Exposure	Outcomes	Primary Findings
Garcia-Larsen et al., 2017 [20]	Adults aged 15–74 years (*n* = 3202)	Cross-sectional observational study	CRS	Fruit and vegetable intake	CRS prevalence	Higher total fruit intake was associated with lower CRS odds (aOR 0.73, 95% CI 0.55–0.97), whereas potato intake was associated with higher odds (aOR 1.82, 95% CI 1.03–3.23); tropical fruit intake was not significant after adjustment (aOR 2.50, 95% CI 0.91–6.92).
Philpott et al., 2019 [21]	Adults (*n* = 873; 402 CRSwNPs, 336 CRSsNPs, 25 AFRS, 110 controls)	Prospective, questionnaire-based, case–control study	CRSwNPs, CRSsNPs, AFRS	Salicylate content in foods	Food-triggered symptom exacerbation; dietary salicylate sensitivity	Dietary salicylate sensitivity was associated with higher odds in CRSwNP (aOR 2.56, 95% CI 1.39–4.71; *p* = 0.002) and CRSsNP (aOR 1.86, 95% CI 1.05–3.30; *p* = 0.034). Wine-triggered symptom exacerbation was significantly more common in CRSwNP and AFRS than controls (*p* < 0.001), while nut-triggered symptoms were more common in AFRS than controls (*p* = 0.001).
Chen et al., 2024 [22]	Adolescents and adults (*n* = 6342; 488 adolescents, 5854 adults)	Cross-sectional observational study	Chronic sinusitis	Dietary intake (fat, energy, carbohydrates, etc.), DII scores	Chronis sinusitis prevalence	Higher dietary fat (*p* = 0.001) and energy intake (*p* = 0.004) were associated with increased CRS prevalence. Participants in the highest DII quartile had increased odds of CRS compared with the lowest quartile (OR 1.48, 95% CI 1.13–1.95; *p* = 0.004).
Han et al., 2024 [23]	Adults (*n* = 48,515; 45,371 control, 3144 CRS)	Cross-sectional observational study	CRS	Frequency of AFH meals	CRS prevalence; persistent olfactory dysfunction; EPOS 2020 [4] endoscopic findings	Higher frequency of AFH meals was associated with increased odds of CRS (1–4 meals/week: aOR 1.14, 95% CI 1.03–1.26; *p* = 0.0119; ≥5 meals/week: aOR 1.21, 95% CI 1.08–1.36; *p* = 0.0013). Frequent AFH meals were also associated with persistent olfactory dysfunction (1–4 meals/week: aOR 1.72, 95% CI 1.17–2.52; *p* = 0.0058; ≥5 meals/week: aOR 2.16, 95% CI 1.46–3.22; *p* = 0.0001), but not with nasal obstruction, nasal discharge, facial pain, middle meatus discharge, or nasal polyps.
Pazdro-Zastawny et al., 2024 [24]	Children and adolescents aged 6–17 years (*n* = 2458)	Cross-sectional observational study	RS	Dietary habits (Fruit, milk, salty snack, and carbonated sweet drink consumption)	RS prevalence	Low fruit intake (<5–6 times/week) was independently associated with increased odds of RS (OR 1.75, 95% CI 1.07–2.86; *p* = 0.027), as was frequent salty snack consumption (≥1–2 times/week; OR 1.91, 95% CI 1.14–3.20; *p* = 0.014). Children with RS also consumed more carbonated sweet drinks (*p* = 0.023), although this was not an independent predictor after multivariable adjustment.
Thai et al., 2025 [25]	Adults (*n* = 10,068)	Cross-sectional observational study	Sinusitis	UPF intake	Self-reported sinusitis; dysgeusia; hyposmia; allergic nasal congestion; allergies; hay fever; serum total IgE	Highest UPF intake quartile was associated with increased odds of sinusitis (adjusted OR 1.54, *p* = 0.007) and dysgeusia (adjusted OR 1.79, *p* = 0.02), with no significant associations observed for hyposmia (*p* = 0.48), allergic nasal congestion (*p* = 0.10), allergies (*p* = 0.88), hay fever (*p* = 0.79), or serum total IgE (*p* = 0.17).

RS, rhinosinusitis; CRS, chronic rhinosinusitis; CRSwNP, chronic rhinosinusitis with nasal polyps; CRSsNP, chronic rhinosinusitis without nasal polyps; AFRS, allergic fungal rhinosinusitis; DII, Dietary Inflammatory Index; AFH, away-from-home; UPF, ultra-processed food; EPOS, European Position Paper on Rhinosinusitis and Nasal Polyps; IgE, immunoglobulin E; aOR, adjusted odds ratio; OR, odds ratio; CI, confidence interval.

**Table 3:** Risk of bias assessment of included cross-sectional studies [20,22,23,24,25] using the JBI Critical Appraisal Checklist for Analytical Cross-Sectional Studies [14].

Checklist Questions	Garcia-Larsen et al., 2017 [20]	Chen et al., 2024 [22]	Han et al., 2024 [23]	Pazdro-Zastawny et al., 2024 [24]	Thai et al., 2025 [25]
Were the criteria for inclusion in the sample clearly defined?	Yes	Yes	Yes	Yes	Yes
Were the study subjects and the setting described in detail?	Yes	Yes	Yes	Yes	Yes
Was the exposure measured in a valid and reliable way?	Yes	Yes	Unclear	Unclear	Yes
Were objective, standard criteria used for measurement of the condition?	Yes	Yes	Yes	Unclear	**No**
Were confounding factors identified?	Yes	Yes	Yes	Yes	Yes
Were strategies to deal with confounding factors stated?	Yes	Yes	Yes	Yes	Yes
Were the outcomes measured in a valid and reliable way?	Unclear	Yes	Yes	Unclear	Unclear
Was appropriate statistical analysis used?	Yes	Unclear	Yes	Yes	Yes

**Table 4:** Risk of bias assessment of the included case–control study [21] using the JBI Critical Appraisal Checklist for Analytical Case–Control Studies [15].

Checklist Questions	Philpott et al., 2019 [21]
Were the criteria for inclusion in the sample clearly defined?	Yes
Were the study subjects and the setting described in detail?	Yes
Was the exposure measured in a valid and reliable way?	No
Were objective, standard criteria used for measurement of the condition?	Yes
Were confounding factors identified?	Yes
Were strategies to deal with confounding factors stated?	Yes
Were the outcomes measured in a valid and reliable way?	No
Was appropriate statistical analysis used?	Yes

## Data Availability

No new data were created or analyzed in this study. Data sharing is not applicable to this article.

## Supplementary Materials

The following supporting information can be downloaded at: https://www.mdpi.com/article/10.3390/nu18142299/s1, Table S1: Detailed search strategy; Table S2: Detailed ROBINS-I risk-of-bias judgements and supporting rationale by domain for each included study; Table S3: Methodological quality of the included cross-sectional studies, appraised with the JBI Critical Appraisal Checklist for Analytical Cross-Sectional Studies; Table S4: Methodological quality of Philpott et al. (2019), appraised with the JBI Critical Appraisal Checklist for Analytical Case-Control Studies; Table S5: Confounding variables adjusted for in included observational studies.
