# Supplementary material for: Diet, Nutrition, and Rhinosinusitis: A Systematic Review of Dietary Interventions and Exposures
Source: Nutrients. 2026 Jul 14;18(14):2299. doi: 10.3390/nu18142299 (PMC13414780; doi:10.3390/nu18142299)
Supplement: Supplementary file 1 [file nutrients-18-02299-s001.zip › Supplementary Table S1. Search Strategy.pdf]

**Supplementary Table S1.** Detailed search strategy.

| Database | Date Searched | Search Strategy | Limit/Filters Applied |
|---|---|---|---|
| Scopus | June 28, 2026 | ( TITLE-ABS-KEY ( sinusitis OR "acute sinusitis" OR "chronic sinusitis" OR rhinosinusitis OR "chronic rhinosinusitis" OR "acute rhinosinusitis" ) ) AND ( TITLE-ABS-KEY ( diet OR "mediterranean diet" OR nutrition OR "anti-inflammatory diet" OR "food consumption" OR "processed foods" OR "ultra processed foods" OR "food intake" OR "eating habits" ) ) AND ( LIMIT-TO ( EXACTKEYWORD , "Humans" ) ) AND ( LIMIT-TO ( LANGUAGE , "English" ) ) | English language, human subjects, publication date inception-2026 |
| PubMed | June 28, 2026 | ( ( "Sinusitis"[Mesh] OR sinusitis[tiab] OR "acute sinusitis"[tiab] OR "chronic sinusitis"[tiab] OR rhinosinusitis[tiab] OR "acute rhinosinusitis"[tiab] OR "chronic rhinosinusitis"[tiab] OR "Nasal Polyps"[Mesh] OR nasal polyp*[tiab] ) AND ( "Diet"[Mesh] OR "Mediterranean Diet"[Mesh] OR "Nutrition"[Mesh] OR diet*[tiab] OR "mediterranean diet"[tiab] OR nutrition*[tiab] OR "anti inflammatory diet"[tiab] OR "anti-inflammatory diet"[tiab] OR "food consumption"[tiab] OR "food intake"[tiab] OR "processed foods"[tiab] OR "ultra processed foods"[tiab] OR "ultra-processed foods"[tiab] OR "eating habits"[tiab] ) ) | English language, human subjects, publication date inception-2026 |
| Cochrane Library | June 28, 2026 | #1 MeSH descriptor: [Sinusitis] explode all trees<br>#2 MeSH descriptor: [Rhinosinusitis] explode all trees<br>#3 MeSH descriptor: [Rhinitis] explode all trees<br>#4 MeSH descriptor: [Nasal Polyps] explode all trees<br>#5 MeSH descriptor: [Paranasal Sinus Diseases] explode all trees<br>#6 MeSH descriptor: [Nose Diseases] explode all trees<br>#7 Free-text terms: sinusitis OR rhinosinusitis OR rhinitis OR sinonasal OR para nasal OR paranasal OR nasal congestion OR rhinorrhea OR chronic sinusitis OR acute sinusitis OR | English language, human subjects, publication date inception-2026 |

chronic rhinosinusitis OR acute rhinosinusitis OR CRS OR CRSwNP OR CRSsNP OR ARS OR nasal polyp* OR polyposis
#8 #1 OR #2 OR #3 OR #4 OR #5 OR #6 OR #7
#9 MeSH descriptor: [Diet] explode all trees
#10 MeSH descriptor: [Diet Therapy] explode all trees
#11 MeSH descriptor: [Nutrition Therapy] explode all trees
#12 MeSH descriptor: [Food] explode all trees
#13 MeSH descriptor: [Eating] explode all trees
#14 MeSH descriptor: [Feeding Behavior] explode all trees
#15 MeSH descriptor: [Diet, Mediterranean] explode all trees
#16 MeSH descriptor: [Diet, Western] explode all trees
#17 MeSH descriptor: [Diet, Reducing] explode all trees
#18 MeSH descriptor: [Caloric Restriction] explode all trees
#19 MeSH descriptor: [Fasting] explode all trees
#20 MeSH descriptor: [Energy Intake] explode all trees
#21 Free-text terms: diet* OR nutrition* OR food* OR eating OR dietary change* OR dietary modification* OR diet modification* OR dietary intervention* OR dietary pattern* OR diet pattern* OR diet quality OR intake OR consumption OR food intake OR energy intake OR eliminat* OR restrict* OR avoidance OR exclusion OR gluten free OR lactose OR dairy OR low histamine OR fasting OR time restricted OR time-restricted OR caloric restriction OR calorie restriction OR Mediterranean diet OR Western diet OR ketogenic diet OR keto OR low carbohydrate OR low carb OR low fat OR high fat OR high sugar OR high fiber OR high salt OR plant based OR vegetarian OR vegan OR ultra processed OR ultra-processed
#22 #9 OR #10 OR #11 OR #12 OR #13 OR #14 OR #15 OR #16 OR #17 OR #18 OR #19 OR #20 OR #21
#23 #8 AND #22

| | | | |
|---|---|---|---|
| Web of Science | June 28, 2026 | TS=( sinusitis OR "acute sinusitis" OR "chronic sinusitis" OR rhinosinusitis OR "acute rhinosinusitis" OR "chronic rhinosinusitis" OR "nasal polyp*" OR CRS OR CRSwNP OR CRSsNP OR ARS ) AND TS=( diet OR "mediterranean diet" OR nutrition OR "anti-inflammatory diet" OR "anti inflammatory diet" OR "food consumption" OR "food intake" OR "processed foods" OR "ultra processed foods" OR "ultra-processed foods" OR "eating habits" OR dietary OR dietary pattern* OR dietary intervention* ) | English language, human subjects, publication date inception-2026 |
