# Supplementary material for: Diet, Nutrition, and Rhinosinusitis: A Systematic Review of Dietary Interventions and Exposures
Source: Nutrients. 2026 Jul 14;18(14):2299. doi: 10.3390/nu18142299 (PMC13414780; doi:10.3390/nu18142299)
Supplement: Supplementary file 1 [file nutrients-18-02299-s001.zip › Supplementary Table S2. ROBINS-I.pdf]

Supplementary Table S2. Detailed ROBINS-I risk-of-bias judgements and supporting rationale by domain for each included study.

ROBINS-I domain	Catalayud et al., 2017	Sawani et al., 2017	Odat et al., 2017	Stihl et al., 2025
D1: Bias due to confounding	**Serious**<br>Uncontrolled before–after design with no concurrent control group, so dietary effects cannot be separated from age-related immune maturation, regression to the mean, seasonal variation, or changes in parental care.	**Serious**<br>Nonrandomized design with very small groups (8 intervention and 3 control completers), baseline imbalance in SN-5 scores and lipid markers, and likely differential parental motivation to reduce sugar.	**Serious**<br>No concurrent control group while all patients continued nasal steroid spray and saline irrigation, so improvement cannot be attributed to food elimination rather than ongoing therapy, placebo effects, or regression to the mean.	**Serious**<br>Patients self-selected into the dietary, FESS, or dupilumab groups; the dietary group was younger with lower baseline symptom burden and fewer prior surgeries, and no multivariable adjustment was performed because of the small sample.
D2: Bias in selection of participants into the study	**Low**<br>Consecutive eligible patients were enrolled on the basis of prior-year recurrent infection history before the intervention, with little indication of post-intervention selection.	**Moderate**<br>Controls presented for non-nasal complaints whereas the experimental group had chronic nasal symptoms, so the groups were clinically different at baseline and not exchangeable.	**Moderate**<br>Consecutive prospective enrollment reduced selection bias, but inclusion required suggestive food-allergy history and positive sensitization, limiting representativeness.	**Serious**<br>Only patients with good self-reported dietary adherence at both follow-ups who returned for assessment were included in the dietary cohort, enriching it with more motivated patients who were more likely to improve.
D3: Bias in classification of interventions	**Moderate**<br>The Mediterranean diet program was clearly defined, but adherence was behaviour-based and partly captured by questionnaire, allowing some misclassification of actual exposure.	**Moderate**<br>The counseling-based intervention was defined, but sugar reduction relied on parent-reported beverage logs that may not capture total intake, particularly outside the home.	**Moderate**<br>Elimination of sensitized foods was clearly defined, but adherence relied on patient report without objective verification.	**Moderate**<br>Treatment groups were clearly described, but dietary adherence was based on self-report rather than food diaries or objective dietary assessment.
D4: Bias due to deviations from intended interventions	**Moderate**<br>Eight patients withdrew, mostly because of difficulty adhering to the diet, and adherence-related deviations could not be formally addressed in the absence of a control group.	**Moderate**<br>Actual sugar reduction differed markedly between groups (46% versus 11%), and no adjustment for differential adherence or motivation was reported.	**Serious**<br>Two non-adherent participants were excluded, and the analysis was per-protocol rather than intention-to-treat, which likely overestimates treatment benefit.	**Serious**<br>Patients continued topical corticosteroids and were advised to avoid NSAIDs alongside dietary modification, and non-adherent patients were excluded rather than analyzed, so per-protocol inclusion likely overestimates dietary benefit.
D5: Bias due to missing data	**Moderate**<br>Attrition was low at roughly 6% (128 of 136 completed), although some loss was diet-related and could relate to intervention response.	**Serious**<br>Of 17 recruited, only 11 completed both visits (about 35% attrition), largely related to follow-up blood draws, within an already small sample.	**Serious**<br>Six of 22 participants (27%) were excluded from the final analysis (4 lost to follow-up and 2 non-adherent) in a small pilot sample, threatening validity.	**Serious**<br>Many patients offered the dietary intervention did not return for follow-up and were excluded (the exact number was not reported), and non-return may relate to poorer response or adherence.
D6: Bias in measurement of outcomes	**Moderate**<br>Definitions for URTI, acute otitis media, and rhinosinusitis were	**Moderate**<br>The SN-5 is a validated instrument, and cytokines were measured	**Moderate**<br>Lund–Kennedy symptom and endoscopic scores are established	**Moderate**<br>SNOT-22, VAS, endoscopy, and BSIT are validated measures, but neither

ROBINS-I domain	Catalayud et al., 2017	Sawani et al., 2017	Odat et al., 2017	Stihl et al., 2025
	described, but the same pediatrician delivered and assessed the intervention without blinding, and several outcomes were subjective.	objectively, but the primary sinonasal symptom outcome was parent-reported and unblinded.	measures, but the study was explicitly open-label with no blinding of participants or assessors.	participants nor clinicians were blinded, leaving the subjective outcomes prone to expectation bias.
D7: Bias in selection of the reported result	**Moderate**<br>Many outcomes were assessed, and no protocol or statistical analysis plan was available, although there was no direct evidence of selective reporting.	**Moderate**<br>Multiple SN-5 domains, cytokines, lipids, glucose, and sugar intake were assessed without an available protocol to confirm a prespecified outcome hierarchy.	**Moderate**<br>Multiple symptom domains and repeated follow-up assessments were analyzed without an available protocol to exclude selective reporting.	**Moderate**<br>Multiple outcomes were assessed at several time points with no available protocol or analysis plan to confirm prespecified outcome selection.
**Overall**	**Serious**<br>Serious risk of bias, driven by serious confounding inherent to the uncontrolled before-after design.	**Serious**<br>Serious risk of bias, driven primarily by uncontrolled confounding and missing data in a very small nonrandomized pilot.	**Serious**<br>Serious risk of bias due to uncontrolled confounding, per-protocol exclusion of non-adherent participants, and 27% attrition without a control group.	**Serious**<br>Serious risk of bias driven by nonrandomized treatment selection, inclusion of only adherent dietary participants, missing follow-up data, absence of multivariable adjustment, and subjective unblinded outcomes.

ROBINS-I, Risk Of Bias In Non-randomized Studies of Interventions; AERD, aspirin-exacerbated respiratory disease; BSIT, Brief Smell Identification Test; CRSwNP, chronic rhinosinusitis with nasal polyps; FESS, functional endoscopic sinus surgery; NSAID, nonsteroidal anti-inflammatory drug; SN-5, Sino-Nasal 5 survey; SNOT-22, 22-item Sino-Nasal Outcome Test; URTI, upper respiratory tract infection; VAS, visual analogue scale.
