# Supplementary material for: Diet, Nutrition, and Rhinosinusitis: A Systematic Review of Dietary Interventions and Exposures
Source: Nutrients. 2026 Jul 14;18(14):2299. doi: 10.3390/nu18142299 (PMC13414780; doi:10.3390/nu18142299)
Supplement: Supplementary file 1 [file nutrients-18-02299-s001.zip › Supplementary Table S3. JBI cross-sectional.pdf]

Supplementary Table S3. Methodological quality of the included cross-sectional studies, appraised with the JBI critical appraisal checklist for analytical cross-sectional studies.

JBI Checklist Question	Garcia-Larsen et al., 2017	Chen et al., 2024	Han et al., 2024	Pazdro-Zastawny et al., 2024	Thai et al., 2025
Were the criteria for inclusion in the sample clearly defined?	**Yes**<br>Inclusion criteria, sampling strategy, and eligibility criteria were clearly described.	**Yes**<br>Participants were selected from the 2012 KNHANES using predefined eligibility criteria, with clear inclusion and exclusion procedures.	**Yes**<br>Participants were selected from KNHANES 2010-2019 using predefined eligibility and exclusion criteria, including exclusion of individuals <18 years, those with missing data, and those with nasal tumors.	**Yes**<br>Inclusion criteria (children aged 6-17 years attending selected public schools) and eligibility were clearly described.	**Yes**<br>Participants were selected from three NHANES cycles using predefined eligibility criteria, with exclusions for missing dietary recall and sinusitis data clearly described.
Were the study subjects and the setting described in detail?	**Yes**<br>Participants were recruited from multiple European countries through the GA²LEN network, and demographic characteristics were comprehensively reported.	**Yes**<br>The source population, recruitment, demographics, and survey setting were comprehensively described.	**Yes**<br>The study population, nationwide sampling framework, recruitment procedures, and participant characteristics were comprehensively described.	**Yes**<br>The study population, recruitment strategy, geographic setting, study period, and participant characteristics were adequately reported.	**Yes**<br>The nationally representative U.S. population, NHANES sampling design, participant characteristics, and study setting were comprehensively described.
Was the exposure measured in a valid and reliable way?	**Yes**<br>Dietary intake was assessed using the GA²LEN Food Frequency Questionnaire, an internationally standardized and previously validated FFQ translated into multiple languages.	**Yes**<br>Dietary intake was obtained using standardized KNHANES 24-hour dietary recall and validated food-frequency questionnaires administered by trained personnel.	**Unclear**<br>Frequency of AFH meals was assessed using a standardized questionnaire; however, it relied on self-report over the previous year, which is susceptible to recall bias and did not capture dietary composition or nutrient intake.	**Unclear**<br>Dietary habits and physical activity were obtained from a parent-/adolescent-completed questionnaire that was developed specifically for the study. No evidence of questionnaire validation or reliability testing was reported, making exposure misclassification possible.	**Yes**<br>UPF intake was quantified using two standardized 24-hour dietary recalls and classified according to the validated NOVA classification system, a widely accepted approach in nutritional epidemiology.
Were objective, standard criteria used for measurement of the condition?	**Yes**<br>CRS was defined using EP³OS criteria, a standardized and validated diagnostic definition used in epidemiologic studies.	**Yes**<br>CRS was identified through the KNHANES ENT examination using physician assessment, nasal endoscopy, and standardized survey procedures.	**Yes**<br>CRS was assessed using physician-administered questionnaires, standardized nasal endoscopy, and EPOS-based criteria by trained otolaryngology physicians.	**Unclear**<br>Although the authors state that rhinosinusitis was physician diagnosed, the diagnosis for this survey relied on parent-reported questionnaire responses, and the authors acknowledge that endoscopy, CT imaging, and medication data were unavailable. They specifically identify self-reported symptoms as a limitation.	**No**<br>Sinusitis was determined by self-report ("Have you ever been told you have sinusitis?") rather than physician examination, validated diagnostic criteria, endoscopy, or imaging, creating potential outcome misclassification.
Were confounding factors identified?	**Yes**<br>The authors identified numerous potential confounders, including age,	**Yes**<br>Important demographic and socioeconomic confounders	**Yes**<br>The study identified multiple demographic and lifestyle confounders, including age, sex,	**Yes**<br>Potential confounders including age, sex, BMI, physical activity, dietary	**Yes**<br>Important demographic, socioeconomic, lifestyle, and medical confounders (age, sex,

JBI Checklist Question	Garcia-Larsen et al., 2017	Chen et al., 2024	Han et al., 2024	Pazdro-Zastawny et al., 2024	Thai et al., 2025
	sex, BMI, smoking, education, employment, supplement use, and total energy intake.	were identified before multivariable analysis.	residence, education, income, occupation, smoking, alcohol use, and obesity.	behaviors, and comorbidities were identified.	race/ethnicity, education, income, obesity, smoking, asthma, emphysema, chronic bronchitis) were identified.
Were strategies to deal with confounding factors stated?	**Yes** Multivariable regression models with adjustment for prespecified confounders and weighting for sampling probability were performed.	**Yes** Multivariable logistic regression was performed adjusting for prespecified confounders.	**Yes** Multivariable logistic regression models adjusted for prespecified confounders were performed.	**Yes** Multivariable logistic regression was performed to identify independent predictors after adjustment for covariates.	**Yes** Multivariable logistic regression adjusting for prespecified confounders was performed. Survey weights and the complex NHANES sampling design were appropriately incorporated.
Were the outcomes measured in a valid and reliable way?	**Unclear** CRS was defined using validated symptom-based EP³OS criteria; however, the diagnosis was based on self-reported questionnaire responses rather than physician examination, nasal endoscopy, or imaging, introducing potential outcome misclassification.	**Yes** CRS diagnosis was based on physician-administered ENT assessments and nasal endoscopy rather than self-report alone.	**Yes** Outcomes included physician-assessed CRS diagnosis, standardized symptom assessment, nasal endoscopy, and objective IgE measurements in subsets of participants.	**Unclear** Outcome ascertainment depended largely on questionnaire-derived information rather than objective clinical verification for all participants, introducing potential information bias.	**Unclear** Outcomes relied on participant self-report without objective confirmation. While the same standardized NHANES questionnaire was used for all participants, recall bias and nondifferential misclassification remain possible. The authors acknowledge this limitation.
Was appropriate statistical analysis used?	**Yes** Statistical methods were robust and included weighted multivariable regression, meta-analysis across centers, assessment of heterogeneity, and correction for multiple testing using the Simes procedure.	**Unclear** Although appropriate regression analyses were performed, the authors replaced missing values for household income and education with the series mean, excluded nearly half of participants from food-frequency analyses because of nonresponse (3,025/6,342), and did not account for the complex KNHANES survey design using sampling weights, which may introduce bias.	**Yes** Appropriate univariable and multivariable logistic regression analyses were conducted with adjusted odds ratios and confidence intervals reported.	**Yes** Appropriate descriptive statistics and multivariable logistic regression were used, with odds ratios, confidence intervals, and significance testing reported.	**Yes** Appropriate survey-weighted analyses, multivariable logistic regression, adjusted odds ratios, confidence intervals, and significance testing were used.

AFH, away-from-home; BMI, body mass index; CI, confidence interval; CRS, chronic rhinosinusitis; CRSwNP, chronic rhinosinusitis with nasal polyps; CT, computed tomography; ENT, ear, nose, and throat; EP³OS, European Position Paper on Rhinosinusitis and Nasal Polyps; EPOS, European Position Paper on Rhinosinusitis and Nasal Polyps; FFQ, food frequency questionnaire; GA²LEN, Global Allergy and Asthma European Network; IgE, immunoglobulin E; JBI, Joanna Briggs Institute; KNHANES, Korea National Health and Nutrition Examination Survey; NHANES, National Health and Nutrition Examination Survey; NOVA, food classification system based on the nature, extent, and purpose of industrial food processing; OR, odds ratio; UPF, ultra-processed food.
