# Supplementary material for: Diet, Nutrition, and Rhinosinusitis: A Systematic Review of Dietary Interventions and Exposures
Source: Nutrients. 2026 Jul 14;18(14):2299. doi: 10.3390/nu18142299 (PMC13414780; doi:10.3390/nu18142299)
Supplement: Supplementary file 1 [file nutrients-18-02299-s001.zip › Supplementary Table S4. JBI case-control.pdf]

Supplementary Table S4. Methodological quality of Philpott et al. (2019), appraised with the JBI critical appraisal checklist for analytical case-control studies.

**JBI Checklist Question**	**Philpott et al., 2019**
Were the criteria for inclusion in the sample clearly defined?	**Yes**<br>Detailed inclusion and exclusion criteria for CRS phenotypes and controls based on EPOS criteria were provided.
Were the study subjects and the setting described in detail?	**Yes**<br>Recruitment from 30 UK tertiary/secondary centers and participant characteristics were clearly described.
Was the exposure measured in a valid and reliable way?	**No**<br>Dietary salicylate exposure was assessed using a non-validated self-administered questionnaire asking whether participants had ever experienced symptoms after consuming broad food groups. No food diary, dietary recall, food frequency questionnaire, or objective assessment of salicylate intake was used. The authors explicitly acknowledge this limitation.
Were objective, standard criteria used for measurement of the condition?	**Yes**<br>CRS phenotypes were diagnosed using established EPOS criteria, with AFRS classified using accepted diagnostic criteria.
Were confounding factors identified?	**Yes**<br>Age, sex, asthma, aspirin sensitivity, autoimmune disease, immunodeficiency, and ciliary dyskinesia were considered.
Were strategies to deal with confounding factors stated?	**Yes**<br>Multivariable logistic regression and sensitivity analyses were performed.
Were the outcomes measured in a valid and reliable way?	**No**<br>Outcomes were based on self-reported food-triggered symptom exacerbation, not objective confirmation through food challenge, allergy testing, or biomarker assessment. Recall bias and misclassification are possible, and the authors acknowledge these limitations.
Was appropriate statistical analysis used?	**Yes**<br>Appropriate regression analyses, adjustment for confounders, confidence intervals, and Bonferroni correction for multiple comparisons were applied.

AFRS, allergic fungal rhinosinusitis; CI, confidence interval; CRS, chronic rhinosinusitis; CRSwNP, chronic rhinosinusitis with nasal polyps; CRSsNP, chronic rhinosinusitis without nasal polyps; EPOS, European Position Paper on Rhinosinusitis and Nasal Polyps; JBI, Joanna Briggs Institute; OR, odds ratio; UK, United Kingdom.
