# Supplementary material for: Diet, Nutrition, and Rhinosinusitis: A Systematic Review of Dietary Interventions and Exposures
Source: Nutrients. 2026 Jul 14;18(14):2299. doi: 10.3390/nu18142299 (PMC13414780; doi:10.3390/nu18142299)
Supplement: Supplementary file 1 [file nutrients-18-02299-s001.zip › Supplementary Table S5. Counfounding Variables Adjusted.pdf]

Supplementary Table S5. Confounding Variables Adjusted for in Included Observational Studies

Study	Adjusted Confounders
Garcia-Larsen et al., 2017	Age, sex, BMI, smoking status, education, employment status, nutritional supplement use, and total energy intake.
Philpott et al., 2019	Age, sex, asthma, and aspirin sensitivity.
Chen et al., 2024	Sex, age, annual household income, occupation, education, and place of residence.
Han et al., 2024	Age, sex, residence, education level, household income, occupation, drinking status, smoking status, and obesity (BMI).
Pazdro-Zastawny et al., 2024	Age, physical education attendance, fruit consumption, and salty snack consumption.
Thai et al., 2025	Age, sex, race/ethnicity, education, income poverty ratio, asthma, overweight status, smoking, emphysema, and chronic bronchitis..
